# Supplementary material for: Use of Specific T Lymphocytes in Treating Cytomegalovirus Infection in Hematopoietic Cell Transplant Recipients: A Systematic Review
Source: Pharmaceutics. 2024 Oct 11;16(10):1321. doi: 10.3390/pharmaceutics16101321 (PMC11510890; doi:10.3390/pharmaceutics16101321)
Supplement: Supplementary file 1 [file pharmaceutics-16-01321-s001.zip › pharmaceutics-3185071-supplementary.pdf]

pharmaceutics

MDPI

Systematic Review

Supplementary Materials: Use of Specific T Lymphocytes in Treating Cytomegalovirus Infection in Hematopoietic Cell Transplant Recipients: A Systematic Review

Tayná F G S Bandeira, Luciana C Marti, Edna T Rother, Lucas Reis Correia and Clarisse M Machado

Table S1. Search strategy.

Database	Search strategy	Date (Results)
PubMed (Medline)	((("Bone Marrow Transplantation"[MeSH Terms] OR "Bone Marrow Transplantation"[Text Word] OR "BMT"[Text Word] OR "Hematopoietic Stem Cell Transplantation"[MeSH Terms] OR "Hematopoietic Stem Cell"[Text Word] OR "HSCT"[Text Word] OR "bone marrow cell transf*"[Text Word] OR "bone marrow graft*"[Text Word]) AND "tissue transplant*"[All Fields]) OR "cell transplant*"[All Fields]) AND ("Cytomegalovirus infections"[MeSH Terms] OR "Cytomegalovirus"[MeSH Terms] OR "cmv infection*"[Text Word] OR "cytomegalovirus infection*"[Text Word] OR "cytomegalovirus disease*"[Text Word] OR "cmv disease*"[Text Word] OR "cytomegalic inclusion disease*"[Text Word] OR "severe cytomegalovirus infection*"[Text Word] OR "severe cmv infection*"[Text Word] OR "Cytomegalovirus reactivation"[Text Word] OR "cs cmv infection*"[Text Word] OR "CS-CMVi"[Text Word] OR "Cytomegalovirus"[Text Word]) AND ("Lymphocyte Transfusion"[MeSH Terms] OR "Lymphocyte infusion"[Text Word] OR "Cellular therapy"[Text Word] OR "immunotherapy, adoptive"[MeSH Terms] OR "Adoptive immunotherapy"[Text Word] OR "adoptive T-cell therapy"[Text Word] OR "ATCT"[Text Word] OR "lymphoproliferation"[Text Word] OR "CMV specific immunity"[Text Word])	06/28/23 (301)<br><br>Update 07/17/24 (14)
Embase	('bone marrow transplantation'/mj/exp OR 'bone marrow transplantation' OR 'hematopoietic stem cell transplantation'/mj/exp OR 'hematopoietic stem cell transplantation') AND ('cytomegalovirus infection'/mj OR 'cytomegalovirus reactivation':ti,ab,kw OR 'cs-cmv infection':ti,ab,kw OR 'cs-cmvi':ti,ab,kw) AND ('lymphocyte transfusion'/mj OR 'cell therapy'/mj OR 'donor lymphocyte infusion'/mj OR 'adoptive immunotherapy'/mj OR 'adoptive t cell therapy'/mj OR 'lymphocyte proliferation'/exp OR 'lymphocyte proliferation' OR 'cmv specific immunity':ti,ab,kw OR 'lymphoproliferation':ti,ab,kw)	06/22/23 (80)<br><br>Update 07/17/2024 (304)

Scopus	(TITLE-ABS-KEY ("Cytomegalovirus infection*") OR TITLE-ABS-KEY ("CMV infection*") OR TITLE-ABS-KEY ("Cytomegalovirus inclusion disease") OR TITLE-ABS-KEY ("CMV inclusion disease*") OR TITLE-ABS-KEY ("Cytomegalic inclusion disease*") OR TITLE-ABS-KEY ("Severe cytomegalovirus infection*") OR TITLE-ABS-KEY ("Severe CMV infection*") OR TITLE-ABS-KEY ("Cytomegalovirus reactivation") OR TITLE-ABS-KEY ("CS-CMV infection*") OR TITLE-ABS-KEY ("CS-CMVi") OR TITLE-ABS-KEY ("Cytomegalovirus")) AND (TITLE-ABS-KEY ("lymphocyte transfusion") OR TITLE-ABS-KEY ("cellular therapy") OR TITLE-ABS-KEY ("immunotherapy, adoptive") OR TITLE-ABS-KEY ("adoptive immunotherapy") OR TITLE-ABS-KEY ("adoptive tcell therapy") OR TITLE-ABS-KEY ("atct") OR TITLE-ABS-KEY ("lymphoproliferation") OR TITLE-ABS-KEY ("lymphoproliferation")) AND (TITLE-ABS-KEY ("bone marrow transplantation") OR TITLE-ABS-KEY ("hematopoietic stem cell transplantation") OR TITLEABS-KEY ("HSCT") OR TITLE-ABS-KEY ("BMT") OR TITLE-ABS-KEY ("Bone marrow cell transf*") OR TITLE-ABS-KEY ("Bone marrow graft*") OR TITLE-ABS-KEY ("tissue transplant*") OR TITLE-ABS-KEY ("cell transplant*"))	06/19/23 (571)<br><br>Update 07/17/24(79)
Web of Science	("bone marrow transplantation") OR ("hematopoietic stem cell transplantation") OR ("HSCT") OR ("BMT") OR ("Bone marrow cell transf*") OR ("Bone marrow graft*") OR ("tissue transplant*") OR ("cell transplant*") AND ("Cytomegalovirus infection*") OR ("CMV infection*") OR ("Cytomegalovirus inclusion disease") OR ("CMV inclusion disease*") OR ("Cytomegalic inclusion disease*") OR ("Severe cytomegalovirus infection*") OR ("Severe CMV infection*") OR ("Cytomegalovirus") OR ("Cytomegalovirus reactivation") OR ("CS-CMV infection*") OR ("CS-CMVi") AND ("Lymphocyte Transfusion") OR ("Lymphocyte infusion") OR ("Cellular therapy") OR ("Immunotherapy, Adoptive") OR ("Adoptive immunotherapy") OR (“adoptive T-cell therapy”) OR (“atct”) OR ("lymphoproliferation") OR ("CMV specific immunity")	06/19/23 (546)<br><br>Update 07/17/24 (0)

Table S2. Excluded studies.

N⁰	Author, year	Title	Reason for exclusion
1	Jiang et al., 2024	Adoptive therapy with cytomegalovirus-specific cytotoxic T lymphocytes for refractory cytomegalovirus DNAemia and disease after allogeneic haematopoietic stem cell transplantation	Closed access.

2	Shen et al., 2024	Circular mRNA-based TCR-T offers a safe and effective therapeutic strategy for treatment of cytomegalovirus infection	Desing: In vitro study
3	Obermaier et al., 2024	Adenovirus- and cytomegalovirus-specific adoptive T-cell therapy in the context of hematologic cell transplan or HIV infection—A single-center experience	Population: Patients with HIV, did not undergo HSCT (hematopoietic stem cell transplantation).
4	Ma et al., 2024	Adoptive transfer of CMV-specific TCR-T cells for the treatment of CMV infection after haploidentical hematopoietic stem cell transplantation	Type of intervention: Prophy-laxis or prevention of viral infections.
5	Kinoshita et al., 2023	Outcomes following posttransplant virus-specific T-cell therapy in patients with sickle cell disease	Type of intervention: Prophylaxis or prevention of viral infections.
6	Peggs et al., 2023	Immunotherapy with CD25/CD71-allodepleted T cells to improve T-cell reconstitution after matched unrelated donor hematopoietic stem cell transplant: a randomized trial	Population: Patients were not refractory or ineligible.
7	Jalili et al., 2023	Virus-Specific T Cells: Promising Adoptive T Cell Therapy Against Infectious Diseases Following Hematopoietic Stem Cell Transplantation	Publication type: Review article.
8	Motta et al., 2023	Applications of virus-specific T cell therapies post-BMT	Desing: Narrative review
9	Papadopoulou et al., 2023	Pathogen-specific T Cells: Targeting Old Enemies and New Invaders in Transplantation and Beyond	Desing: Narrative review
10	Chen et al., 2023	Another tool against cytomegalovirus after allogeneic hematopoietic cell transplantation	Publication type: Comment
11	Pfeiffer et al., 2023	Posoleucel, an Allogeneic, Off-the-Shelf Multivirus-Specific T-Cell Therapy, for the Treatment of Refractory Viral Infections in the Post-HCT Setting	Type of intervention: multivirus specific T cells.
12	Pei et al., 2022	Adoptive therapy with cytomegalovirus-specific T cells for cytomegalovirus infection after haploidentical stem cell transplantation and factors affecting efficacy	Population: Patients were not refractory or ineligible.
13	Liu et al., 2022	Efficacy of pp65-specific TCR-T cell therapy in treating cytomegalovirus infection after hematopoietic stem cell transplantation	Intervention: TCR-T (T cell receptor cell therapy)
14	Chen et al., 2022	Challenges, Recent Advances and Perspectives in the Treatment of Human Cytomegalovirus Infections	Desing: Narrative review
15	Petitpain et al., 2022	Adoptive immunotherapy with antiviral CTL infusion: French clinical and virological safety survey	Publication type: Abstract

16	Ouellette et al., 2022	Adoptive Immunotherapy for Prophylaxis and Treatment of Cytomegalovirus Infection	Desing: Narrative review
17	Jiang et al., 2022	Third-party CMV- and EBV-specific T-cells for first viral reactivation after allogeneic stem cell transplant	Population: Patients were not refractory or ineligible.
18	Mehdizadeh et al., 2021	Immunotherapy with adoptive cytomegalovirus-specific T cells transfer: Summarizing latest gene engineering techniques	Desing: Narrative review
19	Neill et al., 2021	Cell therapy for cytomegalovirus infection	Desing: Narrative review
20	Pei et al., 2021	Comparison of Transplant Donor and Third-Party Donor Derived CMV-Specific T Cells for CMV Infection after Allogenic Stem Cell Transplantation	Publication type: Abstract
21	Dadwal et al., 2021	Posoleucel (ALVR105), an Off-the-Shelf, Multivirus-Specific T-Cell Therapy, for the Prevention of Viral Infections Post-HCT: Results from an Open-Label Cohort of a Phase 2 Trial	Publication type: Abstract
22	Qi et al., 2021	Steroid-resistant intestinal aGVHD and refractory CMV and EBV infections complicated by haplo-HSCT were successfully rescued by FMT and CTL infusion	Design: Case report
23	Jakharia et al., 2021	CMV Infection in Hematopoietic Stem Cell Transplantation: Prevention and Treatment Strategies	Desing: Narrative review
24	Cao et al., 2021	A Novel CMV-Specific TCR-T Cell Therapy Is Effective and Safe for Refractory CMV Infection after Allogeneic Hematopoietic Stem Cell Transplantation	Publication type: Abstract
25	Basso et al., 2020	Harnessing T Cells to Control Infections After Allogeneic Hematopoietic Stem Cell Transplantation	Desing: Narrative review
26	Park et al., 2020	Successful treatment of refractory CMV colitis after haploidentical HSCT with post-transplant cyclophosphamide using CD45RA+ depleted donor lymphocyte infusion	Design: Case report
27	Uygun et al., 2020	Use of low cell dose for unmanipulated donor lymphocyte for management of cytomegalovirus infection: A single-center experience	Design: Case report
28	Flower et al., 2020	Safety and Efficacy of Virus-Specific Cytotoxic T-Lymphocytes Manufactured By the IFN-g Cytokine Capture System for the Treatment of Refractory Adenovirus, Cytomegalovirus, Epstein Barr Virus, and BK Virus Infections in Children, Adolescents and Young Adults after Allogeneic Hematopoietic Stem Cell Transplantation, Solid	Publication type: Abstract

		Organ Transplantation, or with Primary Immunodeficiency (IND# 17449)	
29	Hakki et al., 2020	Moving Past Ganciclovir and Foscarnet: Advances in CMV Therapy	Desing: Narrative review
30	Shafat et al., 2020	Cellular Therapeutic Approaches to Cytomegalovirus Infection Following Allogeneic Stem Cell Transplantation	Desing: Narrative review
31	Chen et al., 2019	Antiviral agents as therapeutic strategies against cytomegalovirus infections	Publication type: review article
32	Cho et al., 2019	Cytomegalovirus Infections after Hematopoietic Stem Cell Transplantation: Current Status and Future Immunotherapy	Publication type: review article
33	Němečková et al., 2019	Antiviral adoptive immunotherapy using antigen-specific T cells in allogeneic hematopoietic stem cell transplant recipients	Publication type: review article
34	Maffini et al., 2019	An update on the treatment of cytomegalovirus infection after allogeneic hematopoietic stem cell transplantation	Publication type: guideline update – update
35	Ottaviano et al., 2019	Adoptive T Cell Therapy Strategies for Viral Infections in Patients Receiving Haematopoietic Stem Cell Transplantation	Desing: Narrative review
36	Jiang et al., 2019	Pathogen-Specific T Cells Beyond CMV, EBV and Adenovirus	Desing: Narrative review
37	Kaeuferle et al., 2019	Strategies of adoptive T-cell transfer to treat refractory viral infections post allogeneic stem cell transplantation	Desing: Narrative review
38	Harris et al., 2019	Virus-Specific T Cells: Current and Future Use in Primary Immunodeficiency Disorders	Population: patients with primary immunodeficiency
39	Qian et al., 2018	Viral-specific T-cell transfer from HSCT donor for the treatment of viral infections or diseases after HSCT	Desing: Narrative review
40	Lindemann et al., 2018	Adoptive transfer of cellular immunity against cytomegalovirus by virus-specific lymphocytes from a third-party family donor	Publication type: correspondence
41	Barrett et al., 2018	Reprint of: Virus-Specific T Cells: Broadening Applicability	Publication type: reissue
42	Poiret et al. 2018	Cytomegalovirus-Specific CD8+T-Cells with Different T-Cell Receptor Affinities Segregate T-Cell Phenotypes and Correlate With Chronic Graft-Versus-Host Disease in Patients Post-Hematopoietic Stem Cell Transplantation	Type Intervention
43	Meesing et al., 2018	Pharmacological and immunological management of cytomegalovirus infection after solid organ and hematopoietic stem cell transplantation	Publication type: expert Opinion
44	Sutrave et al., 2017	Cellular therapy for multiple pathogen infections after hematopoietic stem cell transplant	Desing: Narrative review

45	Fuji et al., 2017	Cytomegalovirus disease in hematopoietic stem cell transplant patients: current and future therapeutic options	Desing: Narrative review
46	Dave et al., 2017	Virus-Specific T Cells for Hematopoietic Stem Cell Transplantation	Desing: Narrative review
47	Houghtelin et al., 2017	Virus-specific T cells for the immunocompromised patient	Desing: Narrative review
48	Kronfli et al., 2017	Management of cytomegalovirus in hematopoietic stem cell transplant recipients: A review of novel pharmacologic and cellular therapies	Desing: Narrative review
49	Blyth et al., 2013	Moving towards pathogen-specific T cells post-stem cell transplant as standard of care	Publication type: review article
50	Roddie et al., 2017	Immunotherapy for transplantation-associated viral infections	Publication type: series review
51	Kable et al., 2017	Resistant cytomegalovirus disease in kidney-pancreas transplant: Treatment with primed T-cell therapy	Publication type: Abstract
52	Audehm et al., 2017	Specific Adoptive Cellular Immunotherapy in Allogeneic Stem Cell Transplantation	Publication type: review article
53	Tzannou et al., 2017	Off-the-Shelf Virus-Specific T Cells to Treat BK Virus, Human Herpesvirus 6, Cytomegalovirus, Epstein-Barr Virus, and Adenovirus Infections After Allogeneic Hematopoietic Stem-Cell Transplantation	Type of intervention: multivirus specific T cells.
54	Bollard et al., 2016	Adoptive T-cell Therapy for Viral Disease in the Setting of Hematopoietic Cell Transplantation	Publication type
55	Muto et al., 2016	Successful treatment of cytomegalovirus enteritis after unrelated allogeneic stem cell transplantation by the infusion of ex vivo-expanded CD4+ lymphocytes derived from the recipient's peripheral blood donor cells	Design: Case report
56	Maffini et al., 2016	Treatment of CMV infection after allogeneic hematopoietic stem cell transplantation	Publication type: review article
57	Fuji et al., 2016	Immunotherapy for opportunistic infections: Current status and future perspectives	Publication type: review article
58	O'Reilly et al., 2016	Virus-specific T-cell banks for 'off the shelf' adoptive therapy of refractory infections	Publication type: review article
59	El Chaer et al., 2016	How I treat resistant cytomegalovirus infection in hematopoietic cell transplantation recipients	Publication type
60	Spielmann et al., 2016	A single exercise bout enhances the manufacture of viral-specific T-cells from healthy donors: Implications for allogeneic adoptive transfer immunotherapy	Population: Not patients who received bone marrow transplants

61	Locatelli et al., 2016	Cytomegalovirus in hematopoietic stem cell transplant recipients-management of infection	Publication type: expert Opinion
62	Xu et al., 2015	Cytomegalovirus specific cytotoxic T lymphocytes for treatment of refractory cytomegalovirus infection in patients following allogeneic hematopoietic stem cell transplantation	Closed access
63	Hanley et al., 2015	Adoptive immunotherapy with the use of regulatory T cells and virus-specific T cells derived from cord blood	Publication type: review article
64	Maecker-Kolhoff et al., 2015	Broad spectrum antiviral T cells for viral complications after hematopoietic stem cell transplantation	Publication type: editorial
65	Nicholson et al., 2015	Cytomegalovirus-specific T-cell therapies: current status and future prospects	Desing: Narrative review
66	Nishihori et al., 2015	Therapeutic strategies for cytomegalovirus in allogeneic hematopoietic cell transplantation	Publication type: review article
67	Einsele et al., 2015	Immunotherapy for viral and fungal infections	Desing: Narrative review
68	Saglio et al., 2014	The time is now: moving toward virus-specific T cells after allogeneic hematopoietic stem cell transplantation as the standard of care	Desing: Narrative review
69	Hanley et al., 2014	Controlling cytomegalovirus: Helping the immune system take the lead	Publication type: review article
70	Sellar et al., 2014	Therapeutic strategies for cytomegalovirus infection in haematopoietic transplant recipients: A focused update	Publication type: review article
71	Leen et al., 2013	Multicenter study of banked third-party virus-specific T cells to treat severe viral infections after hematopoietic stem cell transplantation	Intervention: Multivirus T cells.
72	Blyth et al., 2013	Donor-derived CMV-specific T cells reduce the requirement for CMV-directed pharmacotherapy after allogeneic stem cell transplantation	Intervention: Prophylaxis
73	Samuel et al., 2013	Successful isolation and expansion of CMV-reactive T cells from G-CSF mobilized donors that retain a strong cytotoxic effector function	Outcomes: in vitro analysis
74	Ramírez et al., 2013	Viral-specific adoptive immunotherapy after allo-SCT: The role of multimer-based selection strategies	Publication type: review article
75	Norkin et al., 2012	Armed Killers Face Off against Cytomegalovirus	Publication type: descriptive article
76	Pagliara et al., 2012	Cytotoxic T lymphocytes for the treatment of viral infections and posttransplant lymphoproliferative disorders in transplant recipients	Design: Narrative review without description of the studies

77	Decot et al., 2012	Anti-viral immunotherapy after allogeneic hematopoietic stem cell transplantation	Publication type: book
78	Fuji et al., 2011	Adoptive immunotherapy with virus-specific T cells	Desing: Narrative review
79	Razonable et al., 2010	Immune-based therapies for cytomegalovirus infection	Publication type: review article
80	Cruz et al., 2010	Adverse events following infusion of T cells for adoptive immunotherapy: A 10-year experience	Population: non-BMT patients
81	Mui et al., 2010	T-cell therapy for cytomegalovirus infection	Desing: Narrative review
82	Peggs et al., 2009	Cytomegalovirus-Specific T Cell Immunotherapy Promotes Restoration of Durable Functional Antiviral Immunity following Allogeneic Stem Cell Transplantation	Intervention: Prophylaxis
83	Tuthill et al., 2009	The prevention and treatment of cytomegalovirus infection in haematopoietic stem cell transplantation	Desing: Narrative review
84	Boeckh et al., 2009	How I treat cytomegalovirus in hematopoietic cell transplant recipients	Publication type
85	Brestrich et al., 2009	Adoptive T-cell therapy of a lung transplanted patient with severe CMV disease and resistance to antiviral therapy: Brief communication	Population: lung transplant patient/Design: case study
86	Peggs et al., 2004	Adoptive T cell immunotherapy for cytomegalovirus	Publication type: expert Opinion
87	Aqui et al., 2008	Post-transplant adoptive T-cell immunotherapy	Publication type: book chapter
88	Leen et al., 2008	Cytotoxic T lymphocytes as immune therapy in haematological practice	Desing: Narrative review
89	Fujita et al., 2008	Adoptive cellular immunotherapy for viral diseases	Publication type: review article
90	Bao et al., 2008	Expansion of cytomegalovirus pp65 and IE-I specific cytotoxic T lymphocytes for cytomegalovirus-specific immunotherapy following allogeneic stem cell transplantation	Outcomes: in vitro analysis
91	Micklethwaite et al., 2008	Prophylactic infusion of cytomegalovirus-specific cytotoxic T lymphocytes stimulated with Ad5f35pp65 gene-modified dendritic cells after allogeneic hemopoietic stem cell transplantation	Intervention: Prophylaxis
92	Cao et al., 2008	Cellular Therapy for Cytomeg-Alovirus or Epstein Barr Virus Reactivation Following Alloge-Neic Stem Cell Transplantation Using Virus-Specific Cytotoxic T Lymphocytes	Publication type: Abstract
93	Einsele et al., 2008	CMV-specific T cell therapy	Design: Narrative review without description of the studies

94	Kapp et al., 2007	Adoptive immunotherapy of HCMV infection	Publication type: review article
95	O'Reilly et al., 2007	Adoptive transfer of antigen-specific T-cells of donor type for immunotherapy of viral infections following allogeneic hematopoietic cell transplants	Outcome: Study focused on data on obtaining lymphocytes - in vitro
96	Grigoleit et al., 2006	Adoptive T lymphocyte therapy to fight cytomegalovirus infection in immunodeficient hosts	Desing: Narrative review
97	Moss et al., 2005	Cellular immunotherapy for viral infection after HSC transplantation	Publication type: review article
98	Perrucio et al., 2005	Transferring functional immune responses to pathogens after haploidentical hematopoietic transplantation.	Intervention: T cells for duovirus
99	Lim et al., 2004	Adoptive immunotherapy for Cytomegalovirus (CMV) disease in immunocompromised patients	Desing: Narrative review
100	Peggs et al., 2004	Adoptive Cellular Therapy for Cytomegalovirus Following Allogeneic Stem Cell Transplantation: Toxicity and Efficacy.	Publication type: Abstract
101	Bollard et al., 2004	Adoptive immunotherapy for posttransplantation viral infections	Does not provide details of outcomes, mentions more about prophylaxis
102	Locatelli et al., 2004	Innovative approaches of adoptive immune cell therapy in paediatric recipients of haematopoietic stem cell transplantation	Desing: Narrative review
103	Simpson et al., 2004	Immunotherapy and gene therapy.	Closed access
104	Peggs et al., 2004	Augmentation of Virus-Specific Immunity After Hematopoietic Stem Cell Transplantation by Adoptive T-Cell Therapy	Population: Patients were not refractory or ineligible. Patients used adoptive therapy even if they had not previously taken antiviral therapy.
105	Peggs et al., 2003	Adoptive cellular therapy for early cytomegalovirus infection after allogeneic stem-cell transplantation with virus-specific T-cell lines	Publication type: research letters
106	Einsele et al., 2003	Antigen-specific T cells for the treatment of infections after transplantation	Publication type: review article
107	Einsele et al., 2003	Immunotherapy of cytomegalovirus infection after stem-cell transplantation: A new option?	Post type: comment
108	Moss et al., 2003	The cellular immunotherapy of viral infection	Publication type: review article
109	Peggs et al., 2002	Clinical trials with CMV-specific T cells	Desing: Narrative review
110	Einsele et al, 2002	Infusion of cytomegalovirus (CMV)-specific T cells for the treatment of CMV	Intervention: Prophylaxis

		infection not responding to antiviral chemotherapy	
111	Khare et al., 2001	Cytomegalovirus treatment options in immunocompromised patients	Publication type: review article
112	Roback et al., 2001	Adoptive immunotherapy using polyclonal allogeneic T-cells with limited GvHD activity protect against lethal post-BMT CMV infections.	Closed access
113	Riddell et al., 2000	Adoptive Immunotherapy of Human Diseases with Antigen-Specific T-Cell Clones	Publication type: book
114	Lucas et al., 2000	A phase I-II trial to examine the toxicity of CMV- and EVB-specific cytotoxic T lymphocytes when used for prophylaxis against EVB and CMV disease in recipients of CD34-selected/T cell-depleted stem cell transplants	Publication type: clinical protocol
115	Einsele et al., 1999	Adoptive immunotherapy to control CMV infection after allo-BMT: Preclinical and clinical results.	Publication type: Abstract
116	Walter et al., 1995	Reconstitution of Cellular Immunity against Cytomegalovirus in Recipients of Allogeneic Bone Marrow by Transfer of T-Cell Clones from the Donor	Intervention: Prophylaxis
117	GREENBERG, P. D. 1991	Development of a Treatment Regimen for Human Cytomegalovirus (CMV) Infection in Bone Marrow Transplantation Recipients by Adoptive Transfer of Donor-Derived CMV-Specific T Cell Clones Expanded In Vitro	Intervention: does not mention adoptive therapy

Table S3. Funding sources for the studies included.

First author, Year	Funding Sources
Bao, L. (2012) [19]	Grants NIH R01CA106319-2 and the Four Diamonds Foundation for Cancer Research
Koehne, G. (2015) [21]	Partial funding for this work was provided by NIH grants NCI CA23766 and NCI R21CA162002 and The Major Family Fund for Cancer Research, Max Cure Fund for Pediatric Cancer Research, Aubrey Fund for Pediatric Cancer Research, and The Claire Tow Chair in Pediatric Oncology Research
Pei, Xu-Ying (2017) [24]	China National Natural Science Foundation and the Beijing Talent Fund, the National China's Key Research and Development Program and the Foundation for Innovative Research Groups of the National Natural Science Foundation of China
Withers, B (2017) [27]	National Council for Health and Medical Research
Neuenhahn, M (2017) [23]	Stage Cell Therapeutics, Juno Cell Therapeutics Inc.
Kállay, K (2018) [22]	Not reported
Tzannou, I. (2019) [25]	Viracyte
Fabrizio, V. A. (2021) [20]	No funding

Ruan, Y. (2022) [26]	No funding
Prockop, S. E. (2023) [28]	Memorial Sloan Kettering Cancer Center
Keller, M. D. (2024) [29]	Dr. Michael Pulsipher

Table S4. Assessment of certainty of evidence—GRADE.

**Certainty assessment**							**Number of patients**		**Effect Estimation**			
**№ of studies**	**Study design**	**Risk of bias**	**Inconsistency**	**Indirect evidence**	**Inaccuracy**	**Other Considerations**	**hCMV-specific T cell adoptive immunotherapy**	**no treatment available**	**Relative (95% CI)**	**Absolute (95% CI)**	**Certainty**	**Importance**
**Security _ GvHD**												
10	non-randomised studies	very serious[a]	not serious	not serious	not serious	none	7 events /228 (3.1%)				⊕⊕◯◯ Low	IMPORTANT
**Response rate, Clearance, or viral load decrease (%)**												
11	non-randomised studies	serious[b]	not serious[c]	not serious	not serious	none	229 events/301 (76%) .				⊕⊕⊕◯ Moderate	CRÍTICO
**Death due to CMV**												
8	non-randomised studies	serious[d]	not serious	not serious	not serious	none	31 events /307 (10.1%)				⊕⊕⊕◯ Moderate	CRÍTICO
**CMV-CS recurrence after AI**												
8	non-randomised studies	very serious[e]	not serious	not serious	not serious	none	51 events /213 (23.9%)				⊕⊕◯◯ Low	IMPORTANTE
**Post-infusion adverse events**												

**Certainty assessment**							**Number of patients**		**Effect Estimation**			
**№ of studies**	**Study design**	**Risk of bias**	**Inconsistency**	**Indirect evidence**	**Inaccuracy**	**Other Considerations**	**hCMV-specific T cell adoptive immunotherapy**	**no treatment available**	**Relative (95% CI)**	**Absolute (95% CI)**	**Certainty**	**Importance**
9	non-randomised studies	very seriousf	not serious	not serious	not serious	none	9 events/222 (4%)				⊕⊕◯◯ Low	IMPORTANTE
**Cytokine release syndrome**												
8	non-randomised studies	very seriousg	not serious	not serious	not serious	none	2 events /155 (1.2%)				⊕⊕◯◯ Low	IMPORTANTE

Legend: CI: confidence interval.

Explanations.

a. Five studies out of eleven presented serious risk of bias (Bao et al., Koehne et al., Kallay et al., Neuenhahn et al., Tzannou et al., Prockop et al.). One study presented critical risk of bias (Pei et al.). And three studies presented moderate risk of bias (Ruan et al., Withers et al., Keller et al.). The studies were penalized in the confounding factor domain due to the lack of blinding, and as they are single-arm studies they do not present randomization.

b. Six studies out of eleven presented serious risk of bias (Bao et al., Koehne et al., Kallay et al., Neuenhahn et al., Tzannou et al., Prockop et al.). One study presented critical risk of bias (Pei et al.). And four studies presented moderate risk of bias (Ruan et al., Withers et al., Keller et al., Fabrizio et al.). The studies were penalized in the confounding factor domain due to the lack of blinding, and as they are single-arm studies they do not present randomization.

c. Seven studies evaluated the elimination of viral load. The studies had different characteristics in the population studied and different ways of measuring the reported results. The majority showed elimination or decreased levels of viral DNA.

d. Three studies out of eleven presented serious risk of bias (Koehne et al., Kallay et al., Prockop et al.). One study presented critical risk of bias (Pei et al.). And four studies presented moderate risk of bias (Ruan et al., Withers et al., Keller et al., Fabrizio et al.). The studies were penalized in the confounding factor domain due to the lack of blinding, and as they are single-arm studies they do not present randomization.

e. Four studies out of eleven presented serious risk of bias (Bao et al., Koehne et al., Kallay et al., Tzannou et al.). One study presented critical risk of bias (Pei et al.). And three studies presented

moderate risk of bias (Ruan et al., Withers et al., Fabrizio et al.). The studies were penalized in the confounding factor domain due to the lack of blinding, and as they are single-arm studies they do not present randomization.

f. Five studies out of eleven presented serious risk of bias (Koehne et al., Kallay et al., Neuenhahn et al., Tzannou et al., Prockop et al.). One study presented critical risk of bias (Pei et al.). And three studies presented moderate risk of bias (Ruan et al., Withers et al., Keller et al.). The studies were penalized in the confounding factor domain due to the lack of blinding, and as they are single-arm studies they do not present randomization.

g. Four studies out of eleven presented serious risk of bias (Bao et al., Koehne et al., Kallay et al., Neuenhahn et al., Tzannou et al.). One study presented critical risk of bias (Pei et al.). And three studies presented moderate risk of bias (Ruan et al., Withers et al., Keller et al.). The studies were penalized in the confounding factor domain due to the lack of blinding, and as they are single-arm studies they do not present randomization.

Table S5. Summary of Doses, Dosage, and Cell Sources for Included Studies.

First author, Year	Cell origin	Dosage	Number of doses
Bao, L. (2012) [19]	NR	2 patients treated with 2.5 × 10^5 CTL/kg and 5 patients treated with 5 × 10^5 CTL/kg.	2 pts received 2 doses, and 5 pts received one dose.
Koehne, G. (2015) [21]	Primary donor (n = 16) Third party (n = 1)	Group 1 (n = 3) received 5 × 10^5 T cells/kg; Group 2 (n = 4), 1 × 10^6 T cells/kg; Group 3 (n = 3), 2 × 10^6 T cells/kg; Group 4 (n = 6), weekly doses of 1 × 10^6 T cells/kg.	NR
Pei, Xu-Ying (2017) [24]	Third party (n = 32)	NR	17 pts received 1 dose, 14 pts 2 doses, and 1 pt 3 doses.
Withers, B (2017) [27]	Third party	2 × 10^7 cells/m^2 and additional dose of 5 × 10^7 cells/m^2 if infusion criteria were met	14 pts received 1 dose, 10 pts 2 doses, 2 pts 3 doses, and 2 pts 4 doses.
Neuenhahn, M (2017) [23]	(n = 28)	780 cells/kg of body weight–872,000 cells/kg of body weight	17pts received single dose.
Kállay, K (2018) [22]	Third party (n = 17)	9.9 (6.7 − 25) × 10^3/kg CD4+ and a median of 32.6 (16 − 125.1) × 10^3/kg CD8+.	6 pts received single dose.
Tzannou, I. (2019) [25]	Third party	2 × 10^7 VSTs/m^2.	8 pts single dose, 1 pt 2 doses, 1 pt 3 doses
Fabrizio, V. A. (2021) [20]	(3 CMV)	NR	61 pts received 1 dose, 24 pts 2 doses, 10 pts 3 doses, and 9 pts > 4 doses.

Ruan, Y. (2022) [26]	(2 CMV + ADV); (1 CMV + EBV)	Median TNC = 1.8 × 10^8/kg	NR
Prockop, S. E. (2023) [28]	Third party		
Keller, M. D. (2024) [29]	Third party		

Legend: NR: Not reported; TNC: Total nucleated cell; CLT: T lymphocyte cells; CMV: Cytomegalovirus; ADV: Adenovirus EBV: Epstein-Barr virus; Pts: Patients.
